# Functional MRI mapping of visual function and selective attention for performance assessment and presurgical planning using conjunctive visual search

**DOI:** 10.1002/brb3.213

**Published:** 2014-01-19

**Authors:** Jason G Parker, Eric J Zalusky, Cemil Kirbas

**Affiliations:** 1 Research Institute, Wright State University4035 Colonel Glenn Highway, Dayton, Ohio, 45435; 2 Imaging Science Research, Kettering Health Network3535 Southern Blvd., Boonshoft Tower 4th Floor, Kettering, Ohio, 45458; 3 Department of Psychiatry, Wright State University627 S. Edwin C. Moses Blvd., Dayton, Ohio, 45435

**Keywords:** fMRI, magnetic resonance imaging, neurosurgery, occipital lobe, visual function

## Abstract

**Background:**

Accurate mapping of visual function and selective attention using fMRI is important in the study of human performance as well as in presurgical treatment planning of lesions in or near visual centers of the brain. Conjunctive visual search (CVS) is a useful tool for mapping visual function during fMRI because of its greater activation extent compared with high-capacity parallel search processes.

**Aims:**

The purpose of this work was to develop and evaluate a CVS that was capable of generating consistent activation in the basic and higher level visual areas of the brain by using a high number of distractors as well as an optimized contrast condition.

**Materials and methods:**

Images from 10 healthy volunteers were analyzed and brain regions of greatest activation and deactivation were determined using a nonbiased decomposition of the results at the hemisphere, lobe, and gyrus levels. The results were quantified in terms of activation and deactivation extent and mean *z*-statistic.

**Results:**

The proposed CVS was found to generate robust activation of the occipital lobe, as well as regions in the middle frontal gyrus associated with coordinating eye movements and in regions of the insula associated with task-level control and focal attention. As expected, the task demonstrated deactivation patterns commonly implicated in the default-mode network. Further deactivation was noted in the posterior region of the cerebellum, most likely associated with the formation of optimal search strategy.

**Conclusion:**

We believe the task will be useful in studies of visual and selective attention in the neuroscience community as well as in mapping visual function in clinical fMRI.

## Introduction

Visual search (VS) is an important cognitive process used in a variety of operational tasks including the analysis of areal and satellite image data and the examination and interpretation of medical images (Elazary and Itti 2010; Eckstein 2011; Biggs et al. 2013). The ability to map the neural processes involved in VS using functional MRI (fMRI) is useful in the development of methods to assess and augment human performance (Proulx 2011). Accurate mapping of visual function is also of significant importance in neurosurgical treatment planning of lesions in or near the occipital lobe, as well as areas of the parietal and temporal lobes which receive visual information through the dorsal and ventral streams (Roux et al. 2001). However, existing studies of clinical fMRI for presurgical mapping of visual function have focused on passive stimuli based on the perception of flashing lights during scanning (Schulder et al. 1999; Li et al. 2013), creating a need for investigations of new and potentially more robust activation paradigms (Machielsen et al. 2000). The most common task used in fMRI studies of VS is the feature search, a high-capacity parallel search process in which the target can be identified from distractors through features which are readily separable such as color or shape. These separable features are detected in parallel and can often be identified without actually being located (Treisman and Gelade 1980). Neural processing of the feature search task begins with basic visual processing in the occipital lobe and then transfers to a frontoparietal attentional network (Corbetta and Shulman 2002). Recent research into feature search using sophisticated model-based analysis has further identified contributions from specific neural regions in parietal and occipital cortical structures, as well as the temporoparietal junction (TPJ) in the response to the relevant saliency of targets (Mavritsaki et al. 2010).

Conjunctive visual search (CVS) is a low-capacity serial search process in which the search target is defined by two or more unique features. CVS requires conscious processing and engagement of additional higher level neural resources (Kristjansson et al. 2002). The anatomical locations of these additional resources vary to some extent in the literature, with increased activation being found in the superior parietal cortex (Corbetta et al. 1995), a superior region of the frontal cortex associated with working memory (Leonards et al. 2000), and frontoparietal regions that include the frontal eye fields (O'Shea et al. 2006). More generally, conjunction is associated with a significantly higher slope of the search time versus number of distractors curve (Wolfe 1998) compared with feature search, and thus may generate greater activation in basic visual processing regions (Nobre et al. 2003). Furthermore, Kahneman and Henik (1981) have shown that selective attention is impacted by the spatial distribution of objects during VS, and that it is not possible to distribute selective attention over a subset of items which have a random spatial distribution. This work was further confirmed by Treisman (1982), and indicates that the size and shape of the visual attention “spotlight” are constrained (Eriksen and Hoffman 1972). The enhanced activation properties of the CVS are also useful in clinical fMRI for presurgical planning in which already decreased activation and neural function may be present due to necrosis, edema, or tumor mass effect.

Although a number of studies have developed and evaluated CVS tasks for fMRI, the majority have used low numbers of distractors (typically less than 10, maximum of 24). The number of distractors is directly related to task difficulty, and it is of interest to evaluate higher numbers of distractors for mimicking complex and challenging work environments. Furthermore, the majority of previous methods have used contrast conditions that represent different implementations of VS tasks or simply lack visual stimuli. An optimal CVS task for human performance evaluation and clinical fMRI involves a contrast condition which mimics the visual stimuli of the CVS, but does not allow searching. It should be noted that at least one study has attempted to implement such a contrast condition by requiring the subjects to judge the optical density of a single fixation point during the nonsearching condition (Leonards et al. 2000). This approach is effective at stopping searching, but invokes additional neural processes related to contrast perception not necessarily utilized during the searching condition. Finally, in the context of surgical treatment planning, it is also of interest to minimize fast-changing and high-contrast images in the task (e.g., flashing lights), as this may reduce the risk of seizure (Zifkin and Trenite 2000).

The purpose of this work was to develop and evaluate a CVS that was capable of generating consistent activation in the basic and higher level visual areas of the brain by using a high number of distractors as well as an optimized contrast condition. We further sought an implementation that minimized overall image contrast between conditions. Finally, we aimed to fully evaluate the activation and deactivation properties of the task throughout the entire brain.

We developed a CVS based on an array of 60 blue squares and 60 red circles (120 total distractors) in which the task was to identify whether or not the array contained a blue circle. We analyzed images of 10 healthy volunteers scanned using fMRI with the CVS task used for stimulus. After individual and group processing, the resulting data sets were analyzed using a nonbiased ROI approach to determine the regions of greatest activation and deactivation. The activation properties of the new task are presented in terms of activation extent and mean *z*-statistic across three levels of anatomy.

## Materials and Methods

### Participants

Images of 10 healthy, right-handed volunteers were used in this study. The local Institutional Review Board approved the use of the images in this study.

### Task

The CVS task used in this study was based on similar tasks described in the neuropsychological and cognitive science literature (Kristjansson et al. 2002; Shen and Reingold 2003; Muggleton et al. 2008; Saevarsson et al. 2008). The task was implemented in a block design paradigm consisting of two repetitions of an 8-sec rest stimulus followed by two repetitions of an 8-sec task stimulus. Each paradigm thus had 16 sec of rest followed by 16 sec of task for a total paradigm length of 32 sec. The paradigm was repeated 12 times for a total experimental time of 6 min 24 sec.

**Figure 1:**
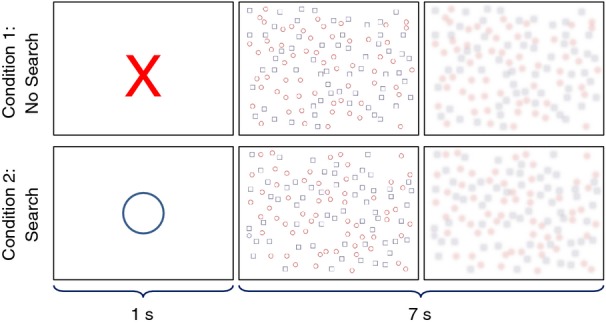
Overview of the conjunctive visual search (CVS) task. Presentation of an X indicates a period without searching, and presentation of an O indicates a period of searching. Upon response, the array is blurred to impair searching.

An overview of the stimuli is shown in Figure 1. The rest stimulus (Condition 1) began with a 1-sec presentation of a single red X centered on the screen. A random array of hollow blue squares and hollow red circles was then presented. Subjects were instructed to immediately click their right index finger upon presentation of the array, causing it to be blurred so that the shapes could no longer be determined. The task stimulus began with a 1-sec presentation of a blue circle centered on the screen. A random array of hollow blue squares and hollow red circles was then presented. Subjects were instructed to determine if the array contained a blue circle and to respond yes (right index finger click) or no (left index finger click) as soon as possible. Upon logging a response, the array was again blurred so that the shapes could not be determined, preventing the subjects from continuing to search. The total time window allotted to view the array and log a response for both rest and task periods was 7 sec.

### Neuroimaging

A 1.5 Tesla (T) MR scanner (Siemens Magnetom Avanto; Siemens, Erlangen, Germany) with an eight-channel birdcage head coil was used for all acquisitions. Participants were positioned on the scanner table supine with their arms at their side and their head stabilized using locking pads attached to the head coil. A video projection system (BrainLogics MRI Digital Projection System; Psychology Software Tools Inc., Sharpsburg, PA) was used for delivery of visual information to a mirror affixed to the top of the head coil. Audio communication with the subject was enabled using noise-canceling headphones. An MR safe vision correction lens system (Psychology Software Tools Inc.) was used to assist patients not able to clearly visualize test letters on the mirror.

After positioning the center of each participant's head at the magnet isocenter, a high-resolution T1-weighted anatomical scan was acquired using a 3D magnetization-prepared rapid acquisition gradient-echo (MPRAGE) sequence with a 512 × 512 element matrix, 120 slices, 1 × 1 × 1 mm voxel size, TR/TE = 500/15 msec, and flip angle = 15°. A single fMRI acquisition was then acquired using a gradient recalled echo sequence with a 64 × 64 element matrix, 24 slices, 4.5 × 4.5 × 5 mm voxel size, 1 mm slice gap, TR/TE = 2000/10 msec, and flip angle = 90°. The stimulus presentation was synced to the pulse sequence using a 5-V transistor-transistor logic pulse received from the imager at the start of every new TR. Consistent with the stimulus outlined in section 2009, 192 volumes were acquired for a total acquisition time of 6 min 24 sec.

### Data processing and analysis

#### Individual image processing

The FMRIB Software Library (Smith et al. 2004; Woolrich et al. 2009) was used for processing of all fMRI data sets. Individual (first-level) analysis was first performed on each of the 4D fMRI data sets. This individual processing began with a high-pass temporal filter with cutoff = 32 sec applied to the 4D fMRI data. Motbion correction was applied by registering each volume to the center volume in the 4D data set by minimizing a correlation ratio cost function with motion estimated based on a rigid-body 12-parameter model (Jenkinson et al. 2002). Spatial smoothing was applied to each volume using a Gaussian convolution with full width half maximum (FWHM) = 5 mm. Low-frequency trends were removed by subtracting a local fit of a straight line across time at each voxel with Gaussian weighting within the line to create a smooth response.

A single explanatory variable (EV) was defined by convolving a boxcar model with 16 sec rest and 16 sec task conditions with a hemodynamic response function modeled by a gamma function with phase offset = 0 sec, standard deviation = 3 sec, and mean lag = 6 sec. The temporal derivative of the original blurred waveform was added to the result to allow for a small shift in phase that could improve the model fit to the measured data. A high-pass temporal filter with cutoff = 32 sec was applied to the model to mimic the processing applied to the measured data. Two contrasts were included in the general linear modeling (GLM): (1) one which applied a weight of +1 to the EV (represented as [+1 0]) and (2) one which applied a weight of −1 to the EV (represented as [−1 0]). These contrasts represented activation (positive correlation with the model) and deactivation (negative correlation with the model), respectively. A GLM with prewhitening was then used to fit the measured data to both model contrasts at each voxel. The resulting *β*-parameter maps were then converted into *z*-statistic maps using standard statistical transforms. To account for false positives due to multiple comparisons, a clustering method was applied in which adjacent voxels with a *z*-statistic of 2.3 or greater were considered a cluster. The significance of each cluster was estimated using Gaussian random field theory and compared to a preselected significance threshold of P < .05. Voxels which did not belong to a cluster or for which the cluster's significance level did not pass the threshold were set to zero. A mean image of the 4D fMRI data was then registered to the individual participants high-resolution anatomical image by minimizing a correlation ratio cost function with motion estimated based on a rigid-body six-parameter model and further registered to the MNI152_T1_2mm_brain template provided in FSL (Collins et al. 1995; Mazziotta et al. 2001) using a 12-parameter model. The transform used to morph the mean fMRI image to the template image was then applied to the *z*-maps so that all statistical volumes were coregistered and in the standard space.

#### Group activation maps

A mean activation map was created for each contrast using a mixed-effects modeling method which was able to carry up variances from the individual analyses to the group analysis (Beckmann et al. 2003). Although less sensitive to group correlations than fixed-effects modeling, this method is advantageous because it allows inferences to be made about the wider populations from which our participants were drawn. The resulting images were thresholded using the clustering method outlined in the Individual analysis section.

#### Temporal characteristics

To investigate the hemodynamic response characteristics of the CVS over the entire paradigm (i.e., 16 sec of rest followed by 16 sec of task), the percentage change in raw gray value from the 4D fMRI data was averaged over all subjects and all paradigm repetitions. This procedure began by first registering the individual 4D fMRI data sets to the standard space using the methods described in the section 1972. A 3D Gaussian convolution of FWHM = 4 mm was then applied to each 4D fMRI volume, followed by four-point linear temporal convolution of weights = [0.25 0.5 0.75 1]. The voxel of greatest significance was identified for each contrast from the group activation maps, and its percentage change was plotted along with the associated standard deviation for a complete paradigm.

#### Quantification of activation and deactivation

To fully evaluate the brain mechanisms associated with performing the CVS, the activation and deactivation properties of the entire brain were quantified using the Talairach coordinate system (Talairach and Tournoux 1988). This procedure began by morphing the activation and deactivation maps for all 10 subjects from the MNI space to the Talairach space using the icbm2tal transform (Lancaster et al. 2007; Laird et al. 2010) provided as a MATLAB (The Mathworks, Natick, MA) m-file on the brainmap.org website (http://www.brainmap.org). The label data and hierarchical list of labels for the Talairach image space (Lancaster et al. 1997, 2000) available on the talairach.org website (http://www.talairach.org) were used to find the voxel extent (number of voxels with *z*-statistic greater than 2.3), mean *z*-statistic, and center of mass (COM) for all combinations of the label hierarchy. This generated 434,371 regions of interest (ROIs) over 7 hemisphere's 12 lobes, 55 gyri, 3 tissue types, and 30 cell types. In an effort to reduce these findings to those of greatest relevance, the data were ordered by extent for both contrasts and the 30 ROIs of greatest extent were tabulated for review.

## Results

**Figure 2:**
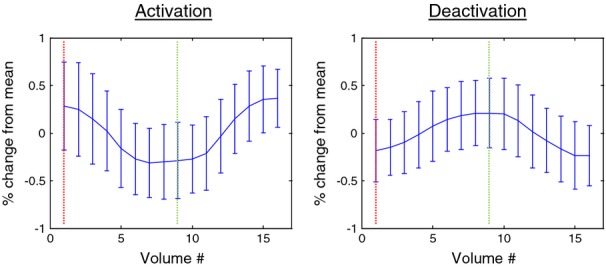
Plots of percent signal change versus volume number averaged over all paradigm iterations and all subjects for the voxel of maximum activation (left) and deactivation (right). The left vertical line indicates onset of the rest stimulus and the right vertical line indicates onset of the conjunctive visual search (CVS) stimulus.

The maximum *z*-statistic for the activation and deactivation contrasts was located in the middle gyrus of the right occipital lobe and the cingulate gyrus in the right limbic lobe, respectively. The time course of these voxels, averaged over all 10 subjects and all 12 paradigm repetitions, is shown in Figure 2 in units of percentage change from the mean gray value. Both voxels demonstrate the expected smooth hemodynamic response. The maximum percentage change for activation occurred 14 sec after onset of the task period and 12 sec after onset of the rest period. The maximum percentage change for deactivation occurred 12 sec after onset of the task period and 14 sec after onset of the rest period.

Table 1 shows the results of the quantitative analysis for the 30 ROIs of greatest activation extent. Activation extent was slightly higher for the right cerebrum than the left. In both sides of the cerebrum, the occipital lobe demonstrated the greatest activation, followed by the frontal and parietal lobes. In both sides of the occipital lobe, the cuneus demonstrated the highest activation, followed by the lingual gyrus and middle occipital gyrus. Activation in the right frontal lobe was concentrated in the middle frontal gyrus, an area housing a large portion of the dorsolateral prefrontal cortex. In the cerebellum, activity was greatest in the posterior lobe, with the declive being the gyrus of highest activation on both sides.

**Table 1:** Extent and mean *z*-statistic for the ROIs of greatest activation.

Level 1 (hemisphere)	Level 2 (lobe)	Level 3 (gyrus)	Extent	Mean *z*
Right cerebrum			84,936	3.59
	Occipital lobe		29,903	3.74
		Cuneus	8748	3.59
		Lingual gyrus	7268	3.67
		Middle occipital gyrus	5768	3.96
	Frontal lobe		20,643	3.42
		Middle frontal gyrus	7343	3.42
		Subgyral	5503	3.45
	Parietal lobe		15,457	3.67
		Precuneus	7781	3.73
	Sublobar		7957	3.49
	Limbic lobe		5875	3.37
	Temporal lobe		4755	3.56
Left cerebrum			70,772	3.58
	Occipital lobe		32,997	3.72
		Cuneus	8817	3.44
		Lingual gyrus	8707	3.71
		Middle occipital gyrus	7747	3.88
	Frontal lobe		14,333	3.47
	Parietal lobe		10,569	3.50
	Sublobar		6207	3.35
Left cerebellum			17,575	3.47
	Posterior lobe		10,849	3.55
		Declive	5291	3.65
	Anterior lobe		6453	3.32
Right cerebellum			14,759	3.50
	Posterior lobe		8336	3.56
		Declive	4699	3.63
	Anterior lobe		6106	3.40
		Culmen	4621	3.44

Table 2 shows the results of the quantitative analysis for the 30 ROIs of greatest deactivation extent. Deactivation extent was higher by nearly a factor of 2 in the left cerebrum compared with the right. In both sides of the cerebrum the frontal lobe demonstrated the greatest deactivation, followed by the parietal, temporal, and limbic lobes. The foci of deactivation within the lobes were not as homogeneous between hemispheres as compared with the activation foci, although the limbic lobe did demonstrate a focus in the cingulate gyrus on both sides.

**Table 2:** Extent and mean *z*-statistic for the ROIs of greatest deactivation.

Level 1 (hemisphere)	Level 2 (lobe)	Level 3 (gyrus)	Extent	Mean *z*
Left cerebrum			84,398	3.59
	Frontal lobe		42,821	3.62
		Superior frontal gyrus	12,572	3.75
		Medial frontal gyrus	12,285	3.77
		Middle frontal gyrus	6490	3.60
		Inferior frontal gyrus	3387	3.29
	Parietal lobe		15,588	3.65
		Inferior parietal lobule	4624	3.52
		Precuneus	4037	3.86
		Supramarginal gyrus	3249	3.56
	Temporal lobe		12,191	3.41
		Middle temporal gyrus	5653	3.38
		Superior temporal gyrus	4092	3.39
	Limbic lobe		10,424	3.67
		Cingulate gyrus	5742	3.73
Right cerebrum			44,173	3.41
	Frontal lobe		14,425	3.35
		Medial frontal gyrus	5430	3.43
		Precentral gyrus	3561	3.21
	Parietal lobe		11,750	3.51
		Inferior parietal lobule	4695	3.38
	Temporal lobe		8418	3.31
		Superior temporal gyrus	3729	3.32
	Limbic lobe		4721	3.55
		Cingulate gyrus	3439	3.63
	Sublobar		3997	3.36
Right cerebellum			7189	3.60
	Posterior lobe		7187	3.60
Left cerebellum			3981	3.37
	Posterior lobe		3971	3.37

**Figure 3:**
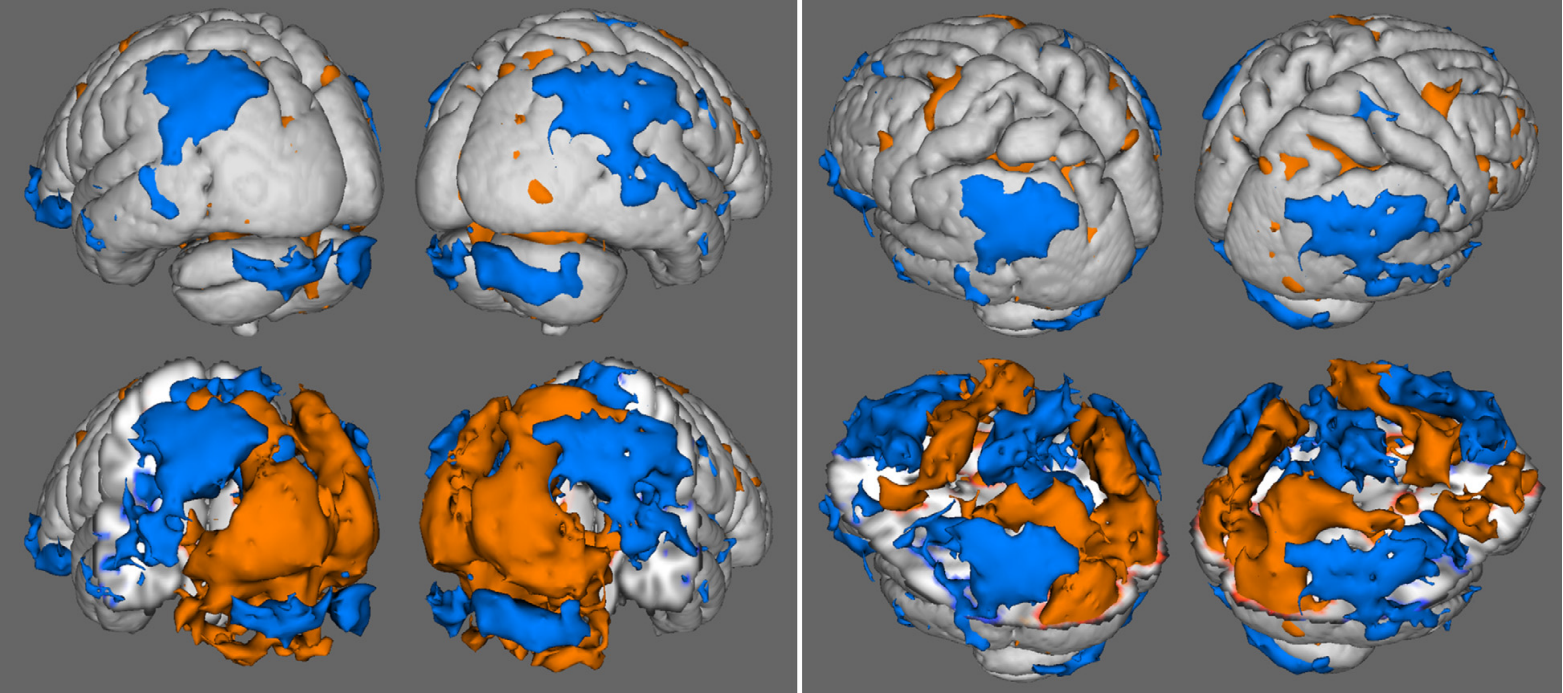
Results of the group maps showing activation (light) and deactivation (dark) for the occipital and cerebellum views (left) and parietal and frontal views (right). Full anatomical surface renderings are shown in the top row, and serve as references for surface renderings in the bottom row which feature anatomy cutouts to reveal deep activation results in regions of interest.

**Figure 4:**
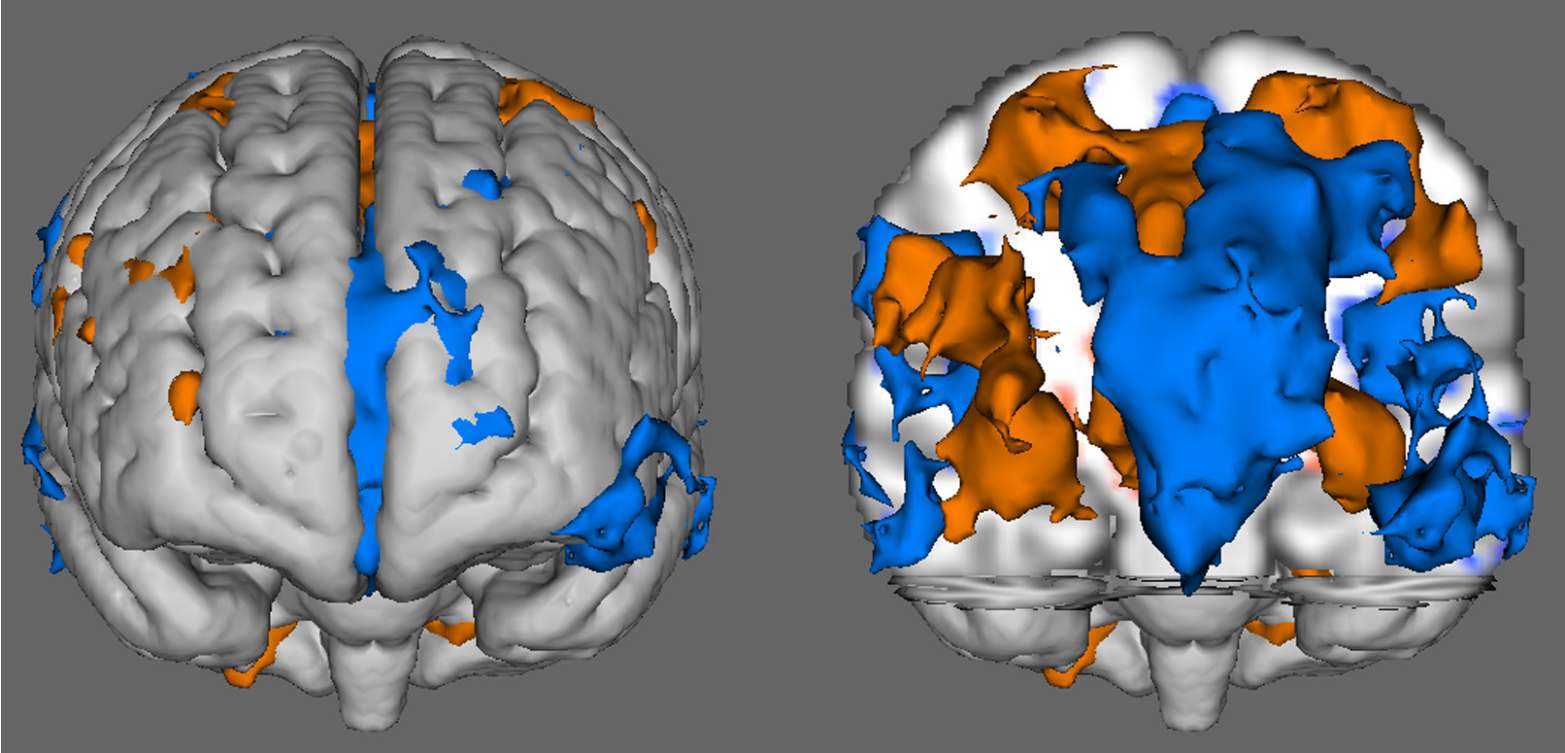
Results of the group map showing activation (light) and deactivation (dark) from the front of the brain.

Volume renderings of the group activation (orange) and deactivation (blue) results are shown in Figure 3. On the left, highly homogenous activation of the occipital lobe is evident, as is activation in the cerebellum. Deactivation is also evident on both sides of the parietal lobe in the angular gyrus, inferior parietal lobule, and precuneus, and extending down into the temporal lobe in the superior temporal gyrus and middle temporal gyrus. On the right, additional activation can be seen in the frontal lobe at the precentral gyrus and medial central gyrus, and transitioning into the cingulate gyrus. Additional deactivation is present throughout the medial frontal gyrus and superior frontal gyrus. Figure 4 also shows activation results in the middle frontal gyrus of the right cerebrum.

**Figure 5:**
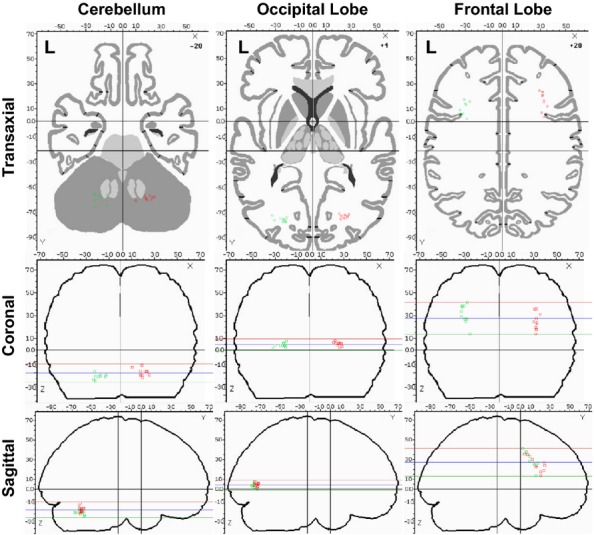
Plots of center of mass (COM) for individual activation results in the cerebellum, occipital lobe, and frontal lobe. The horizontal lines in the coronal and sagittal images represent the top, middle, and bottom of the axial slice.

The spatial distributions of the COMs for the three lobes of greatest extent for both contrasts were plotted using the Brainmap Slueth 2.0 program (Fox and Lancaster 2002; Fox et al. 2005; Laird et al. 2005). The spatial distribution of the activation COMs for the occipital, frontal, and cerebellum is shown in Figure 5, with red indicating the right hemisphere and green indicating the left hemisphere. The cerebellum COMs are grouped near the interface of the posterior and anterior lobes and have similar distributions on left and right sides. The occipital COMs are grouped in the general area of the lingual gyrus and have similar distributions on left and right sides. Finally, the frontal COMs are grouped in the general area of the precentral gyrus and subgyral white matter and demonstrate similar distributions on left and right sides.

**Figure 6:**
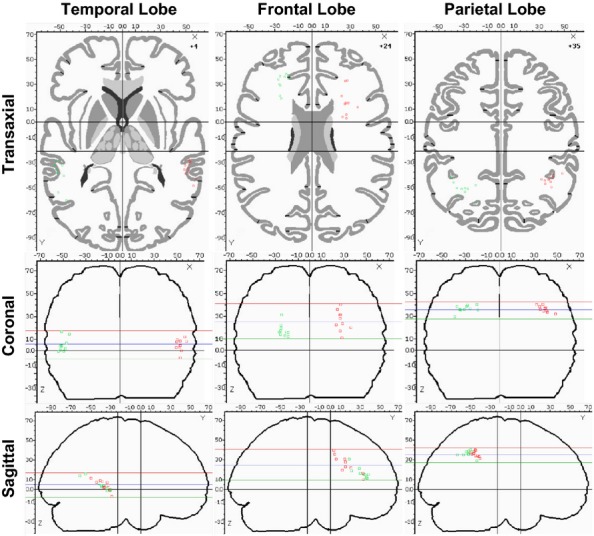
Plots of center of mass (COM) for individual deactivation results in the temporal, frontal, and parietal lobes. The horizontal lines in the coronal and sagittal images represent the top, middle, and bottom of the axial slice.

The spatial distributions of the deactivation COMs for the frontal, parietal, and temporal lobes are shown in Figure 6, with red indicating the right hemisphere and green indicating the left hemisphere. The temporal COMs are grouped near the middle temporal gyrus and subgyral white matter and demonstrate similar distributions on left and right sides. The frontal COMs are grouped in the general area of the anterior cingulate and subgyral white matter, although a broader distribution is seen both in the anterior–posterior direction and the superior–inferior direction. Finally, the parietal COMs are grouped in the general area of subgyral white matter and demonstrate similar distributions on left and right sides.

## Discussion

In this study, the brain mechanisms involved in performing a CVS task developed to map visual and higher level cognitive functions were investigated. The functional relationships between anatomical brain regions identified while performing the task and the cognitive aspects of the task itself are now presented.

### Activation

The task showed consistent and homogenous activation of the occipital lobe, with highest concentrations in the cuneus. This area represents the bulk of the primary visual cortex (Brodmann Area 17) and functionally handles basic visual processing such as spatial frequency, orientation, motion, direction, and speed (Grill-Spector and Malach 2004). The cuneus connects to activation in the precuneus of the parietal lobe via the dorsal stream, which functionally is associated with spatial awareness and representations of object locations (Goodale and Milner 1992; Laycock et al. 2011), and in this case is associated with the perception of the array of shapes during the CVS presentation. The cuneus is also connected to a smaller volume of activation in the inferior temporal cortex of the temporal lobe by activation in the ventral stream, which functionally is associated with object recognition. This activation can be attributed to the recognition of circles against the pattern of squares (Noguchi et al. 2004) as well as the perception of blue and red colors (Zeki 2003). The conjunction task itself is known to require higher level processing that involves all of the primary visual cortex and both the dorsal and ventral streams (Corbetta et al. 1995).

Activation was found in the middle frontal gyrus and subgyral regions of the frontal lobe, most likely associated with the frontal eye fields which are known to play a role in covert VS such as conjunction (Donner et al. 2000). Significant activation was also found in the sublobar areas of the cerebrum which are occupied by the insula. The insula is thought to play a role task-level control and focal attention (Nelson et al. 2010), especially in tasks which may create fatigue or vigilance decrements over time (Eckert et al. 2009), and here most likely is associated with the difficulty of the conjunctive task.

### Deactivation

Deactivation was greatest in the medial, superior, and middle frontal gyri of the frontal lobe. These areas are known to play important roles in core working memory (Boisgueheneuc et al. 2006) and theory of mind (Mason and Just 2009), both of which have been implicated in the default-mode network (DMN) (Garrity et al. 2007). The presence of the DMN activity during baseline is further supported by significant clusters in the cingulate gyrus and precuneus.

### Potential applications in neuroscience research and clinical fMRI

The quantitative control data for the CVS task reported here is intended to provide a foundation for future research into applications of CVS in neuroscience research and surgical planning for tumors in or near visual centers of the brain. In the study of healthy subjects, these findings are useful in contrasting the effects of stimuli (e.g., training, brain stimulation, and endogenous brain control) on baseline activation and regional network components during CVS performance. The findings also provide the ability to quantitatively identify at an individual level, abnormal neuronal and hemodynamic response mechanisms during CVS that may be associated with human performance, potential for response to training, and selection of optimal operators.

Unhealthy populations, such as neurooncology patients, may have abnormal neuronal function and displacement due to tumor mass effect that present significant challenges for comparison to the normative data provided in this study. However, the CVS task tested here may provide an alternative to other visual stimuli used in the surgical planning of tumors in or near the visual centers of the brain that optimizes contrast between stimuli conditions, as well as minimizes the use of rapidly changing brightness levels. The task also demonstrates robust activation of a comprehensive network implicated in visual function and thus may improve the magnitude and extent of activation in clinical studies which can be impacted by patient fatigue and excessive motion (Price et al. 2006). However, demonstration of these proposed benefits in clinical populations requires a randomized controlled trial that is beyond the scope of this study. Therefore, the use of the CVS task or the normative data in unhealthy populations is not supported by the findings of this study alone.

## Conclusion

In this study, we quantitatively analyzed the functional brain properties of a CVS task. The task was found to provide robust activation of the occipital lobe, as well as regions in the middle frontal gyrus associated with coordinating eye movements and in regions of the insula associated with task-level control and focal attention. As expected, the task demonstrated deactivation patterns commonly implicated in the default-mode network. Further deactivation was noted in the posterior region of the cerebellum, most likely associated with the formation of optimal search strategy. We believe the task will be useful in studies of visual attention in the neuroscience community as well as in mapping visual function in clinical fMRI.

## References

[b2] Beckmann CF, Jenkinson M, Smith SM (2003). General multi-level linear modeling for group analysis in FMRI. Neuroimage.

[b3] Biggs AT, Cain MS, Clark K, Darling EF, Mitroff SR (2013). Assessing visual search performance differences between transportation security administration officers and nonprofessional visual searchers. Visual Cogn.

[b4] Boisgueheneuc F, Levy R, Volle E, Seassau M, Duffau H, Kinkingnehun S (2006). Functions of the left superior frontal gyrus in humans: a lesion study. Brain.

[b5] Collins DL, Holmes CJ, Peters TM (1995). Automatic 3-D model-based neuroanatomical segmentation. Hum. Brain Mapp.

[b6] Corbetta M, Shulman GL (2002). Control of goal-directed and stimulus-driven attention in the brain. Nat. Rev. Neurosci.

[b7] Corbetta M, Shulman GL, Miezin FM, Petersen SE (1995). Superior parietal cortex activation during spatial attention shifts and visual feature conjunction. Science.

[b8] Donner TH, Kettermann A, Diesch E, Ostendorf F, Villringer A, Brandt SA (2000). Involvement of the human frontal eye field and multiple parietal areas in covert visual selection during conjunction search. Eur. J. Neurosci.

[b9] Eckert MA, Menon V, Walczak A, Ahlstrom J, Denslow S, Horwitz A (2009). At the heart of the ventral attention system: the right anterior insula. Hum. Brain Mapp.

[b10] Eckstein MP (2011). Visual search: a retrospective. J. Vis.

[b11] Elazary L, Itti L (2010). A Bayesian model for efficient visual search and recognition. Vision Res.

[b12] Eriksen C, Hoffman J (1972). Temporal and spatial characteristics of selective coding from visual displays. Percept. Psychophy.

[b13] Fox PT, Lancaster JL (2002). Mapping context and content: the BrainMap model. Nat. Rev. Neurosci.

[b14] Fox PT, Laird AR, Fox SP, Fox M, Uecker AM, Crank M (2005). Brainmap taxonomy of experimental design: description and evaluation. Hum. Brain Mapp.

[b15] Garrity AG, Pearlson GD, McKiernan K, Lloyd D, Kiehl KA, Calhoun VD (2007). Aberrant “Default Mode” functional connectivity in schizophrenia. Am. J. Psychiatry.

[b16] Goodale MA, Milner AD (1992). Separate visual pathways for perception and action. Trends Neurosci.

[b17] Grill-Spector K, Malach R (2004). The human visual cortex. Annu. Rev. Neurosci.

[b19] Jenkinson M, Bannister PR, Brady JM, Smith SM (2002). Improved optimisation for the robust and accurate linear registration and motion correction of brain images. Neuroimage.

[b20] Kahneman D, Henik A, Kubovy M, Pomerantz JR (1981). Perceptual organization and attention. Perceptual organization.

[b21] Kristjansson A, Wang D, Nakayan K (2002). The role of priming in conjunctive visual search. Cognition.

[b22] Laird AR, Lancaster JL, Fox PT (2005). BrainMap: the social evolution of a functional neuroimaging database. Neuroinformatics.

[b500] Laird AR, Robinson JL, McMillan KM, Tordesillas-Gutierrez D, Moran ST, Gonzales SM, Ray KL, Franklin C, Glahn DC, Fox PT, Lancaster JL (2010). Comparison of the disparity between Talairach and MNI coordinates in functional neuroimaging data: validation of the Lancaster transform. NeuroImage.

[b23] Lancaster JL, Rainey LH, Summerlin JL, Freitas CS, Fox PT, Evans AC (1997). Automated labeling of the human brain: a preliminary report on the development and evaluation of a forward-transform method. Hum. Brain Mapp.

[b24] Lancaster JL, Woldorff MG, Parsons LM, Liotti M, Freitas CS, Rainey L (2000). Automated Talairach Atlas labels for functional brain mapping. Hum. Brain Mapp.

[b25] Lancaster JL, Tordesillas-Gutierrez D, Martinez M, Salinas F, Evans A, Zilles K (2007). Bias between MNI and Talairach coordinates analyzed using the ICBM-152 brain template. Hum. Brain Mapp.

[b26] Laycock R, Cross AJ, Lourenco T, Crewther SG (2011). Dorsal stream involvement in recognition of objects with transient onset but not with ramped onset. Behav. Brain Funct.

[b27] Leonards U, Sunaert S, Van Hecke P, Orban GA (2000). Attention mechanisms in visual search – an fMRI study. J. Cogn. Neurosci.

[b28] Li W, Wait SD, Ogg RJ, Scoggins MA, Zou P, Wheless J (2013). Functional magnetic resonance imaging of the visual cortex performed in children under sedation to assist in presurgical planning. J. Neurosurg. Pediatr.

[b29] Machielsen WCM, Rombouts SARB, Barkhof F, Scheltens P, Witter MP (2000). fMRI of visual encoding: reproducibility of activation. Hum. Brain Mapp.

[b30] Mason R, Just MA (2009).

[b31] Mavritsaki E, Allen HA, Humphreys GW (2010). Decomposing the neural mechanisms of visual search through model-based analysis of fMRI: top-down excitation, active ignoring and the use of saliency by the right TPJ. Neuroimage.

[b32] Mazziotta J, Toga A, Evans A, Fox P, Lancaster J, Zilles K (2001). A probabilistic atlas and reference system for the human brain: International Consortium for Brain Mapping (ICBM). Philos. Trans. R. Soc. B Biol. Sci.

[b33] Muggleton NG, Cowey A, Walsh V (2008). The role of the angular gyrus in visual conjunction search investigated using signal detection analysis and transcranial magnetic stimulation. Neuropsychologia.

[b34] Nelson SM, Dosenbach NUF, Cohen AL, Wheeler ME, Schlaggar BL, Petersen SE (2010). Role of the anterior insula in task-level control and focal attention. Brain Struct. Funct.

[b35] Nobre AC, Coull JT, Walsh V, Frith CD (2003). Brain activations during visual search: contributions of search efficiency versus feature binding. Neuroimage.

[b36] Noguchi Y, Inui K, Kakigi R (2004). Temporal dynamics of neural adaptation effect in the human visual ventral stream. J. Neurosci.

[b501] O'Shea J, Muggleton NG, Cowey A, Walsh V (2006). On the roles of the human frontal eye fields and parietal cortex in visual search. Vis. Cog.

[b37] Price CJ, Crinion J, Friston KJ (2006). Design and analysis of fMRI studies with neurologically impaired patients. J. Magn. Reson. Imaging.

[b38] Proulx MJ (2011). Individual differences and metacognitive knowledge of visual search strategy. PLoS ONE.

[b39] Roux FE, Ibarrola D, Lotterie JA, Chollet F, Berry I (2001). Perimetric visual field and functional MRI correlation: implications for image-guided surgery in occipital brain tumours. J. Neurol. Neurosurg. Psychiatry.

[b40] Saevarsson S, Joelsdottir S, Hjaltason H, Kristjansson A (2008). Repetition of distractor sets improves visual search performance in hemispatial neglect. Neuropsychologia.

[b41] Schulder M, Holodny A, Liu W-C, Gray A, Lange G, Carmel PW (1999). Functional magnetic resonance image-guided surgery of tumors in or near the primary visual cortex. Stereotact. Funct. Neurosurg.

[b42] Shen J, Reingold EM (2003). Guidance of eye movements during conjunctive visual search: the distractor-ratio effect. Can. J. Exp. Psychol.

[b43] Smith SM, Jenkinson M, Woolrich MW, Beckmann CF, Behrens TE, Johansen-Berg H (2004). Advances in functional and structural MR image analysis and implementation as FSL. Neuroimage.

[b44] Talairach J, Tournoux P (1988). Co-planar stereotaxic atlas of the human brain.

[b45] Treisman AM (1982). Preceptual grouping and attention in visual search for features and for objects. J. Exp. Psychol. Human Percept. Perform.

[b46] Treisman AM, Gelade G (1980). A feature-integration theory of attention. Cogn. Psychol.

[b47] Wolfe JM (1998). What can 1 million trials tell us about visual search?. Psychol. Sci.

[b48] Woolrich MW, Jbabdi S, Patenaude B, Chappell M, Makni S, Behrens T (2009). Bayesian analysis of neuroimaging data in FSL. Neuroimage.

[b49] Zeki S (2003). The disunity of consciousness. Trends Cogn. Sci.

[b50] Zifkin BG, Trenite DKN (2000). Reflex epilepsy and reflex seizures of the visual system: a clinical review. Epileptic Disord.

